# Proof of Proficiency of Decentralized Foot-and-Mouth Disease Virus Diagnostics in Germany

**DOI:** 10.3390/v14051098

**Published:** 2022-05-20

**Authors:** Hanna Keck, Bernd Hoffmann, Michael Eschbaumer

**Affiliations:** Institute of Diagnostic Virology, Friedrich-Loeffler-Institut, Federal Research Institute for Animal Health, Suedufer 10, 17493 Greifswald, Germany; hanna.keck@fli.de (H.K.); bernd.hoffmann@fli.de (B.H.)

**Keywords:** foot-and-mouth disease virus, proficiency test, ring trial, diagnostics, real-time RT-PCR, ELISA, exclusion diagnostics

## Abstract

A proficiency test was performed to verify that the regional veterinary laboratories in Germany can provide reliable foot-and-mouth disease virus (FMDV) diagnostics. Overall, 24 samples were to be analyzed for FMDV-specific nucleic acids by real-time RT-PCR, and 16 samples had to be tested by ELISA for antibodies against non-structural proteins of FMDV. For both methods, a range of dilutions of the original materials (inactivated FMDV vaccine or convalescent serum from infected animals, respectively) was prepared, and negative samples were included as well. All 23 participating laboratories were able to detect FMDV genome down to a dilution of 1:100,000 of the vaccine preparation. Even at a dilution of 1:1,000,000, FMDV genome was detected by more than half of the participants. With the antibody ELISA, all sera were correctly identified by all participating laboratories. No false-positive results were returned with either method. All participating laboratories were found to be fully proficient in FMDV diagnostics.

## 1. Introduction

For the first time in history, 10 years have passed without a single case of foot-and-mouth disease (FMD) in the European Union (EU). However, the disease still circulates in countries close to the EU and in many other places in Africa, the Middle East, and Asia [1].

FMD virus (FMDV) is most likely to be spread by the movement of infected animals [2], but the virus can also be transmitted indirectly, with animal products such as milk and meat posing a considerable risk [3]. This was demonstrated by the 2001 outbreak in the United Kingdom, which began in a holding where contaminated food waste of unknown origin was fed to pigs [4]. Apart from animals and their products, the virus can also be spread by contaminated vehicles, clothing, and other fomites [3,5].

Increasing global trade and travel pose a significant risk of reintroduction of FMDV to the EU and Germany. Effective control can only be achieved by early recognition of the disease [5,6,7,8,9]. In the event of an incursion of FMD, its rapid and reliable diagnosis is therefore of paramount importance. There is a concern, however, that clinically unclear cases are not reported by veterinarians or farmers for fear of the possible consequences [10,11,12]. To lower the threshold for these reports and increase diagnostic capacity, a decentralized non-discriminatory exclusion testing scheme has been established in Germany and other countries. Formally declared FMD suspicions must be investigated by an official veterinarian, and samples are sent to the designated national reference laboratory (NRL) as outlined in Article 54 of Regulation (EU) 2016/429 of the European Parliament and of the Council of 9 March 2016 (‘EU Animal Health Law’). In cases that do not rise to the level of an FMD suspicion but where FMD is to be excluded as a possible differential diagnosis, practitioners can submit samples to state laboratories for real-time RT-PCR (RT-qPCR) testing without the involvement of the competent authority and without restrictions placed on the affected farm [13,14,15]. 

Besides virological detection, the tasks of the state laboratories also include serological tests. These do not play a big role in FMD exclusion testing but will be essential if the FMD-free status of Germany is to be regained after an outbreak. In the case of emergency vaccination not followed by slaughter, serological testing of large cohorts of animals must prove that there is no evidence of infection in the vaccinated population [16]. It is therefore critical to reliably differentiate between infected and vaccinated animals (DIVA). 

During the production of DIVA-capable FMD vaccines, the virus is replicated in cell culture, chemically inactivated, and the non-structural proteins (NSP) produced by the infected cells are then removed. In theory, the final formulated vaccine only contains viral capsids (i.e., structural proteins) and denatured viral RNA but no NSP [17]. Vaccinated animals have antibodies only against the structural proteins present in the vaccine. By contrast, infected animals have antibodies against structural proteins but also antibodies against the NSP produced during viral replication in the host. In practice, however, there are two concerns: the NSP removal may be incomplete, leading to eventual seroconversion after repeated vaccinations (risk of false-positive results), and the extent of viral replication in vaccinated animals may be very limited, leading to weak seroconversion and poor clinical sensitivity of the serological tests on the level of an individual animal (risk of false-negative results).

In summary, the state laboratories must be able to perform rapid and reliable virological and serological examinations for FMD, in extreme cases also on a very large scale. At regular intervals, the NRL carries out a ring trial for the state laboratories to confirm their diagnostic proficiency. Each of the 23 participating laboratories was provided 24 samples for analysis by RT-qPCR for FMD-specific nucleic acids and 16 samples for detection of antibodies to non-structural proteins of FMDV by ELISA. The samples were prepared and distributed by the NRL, and the results returned by the participating labs were analyzed and compared to assess their diagnostic capability and the performance of the assays. A high level of diagnostic proficiency must be maintained by the state laboratories to reliably detect a reintroduction of FMD to Germany, effectively contain an outbreak, and quickly regain FMD-free status.

## 2. Materials and Methods

A total of 23 virology panels consisting of 24 samples and 19 serology panels consisting of 16 samples were prepared. The volume of each sample was 500 µL.

For detection of FMDV genome by RT-qPCR, the starting materials were commercial inactivated whole-virus FMD vaccine preparations against several serotypes. The SAT2 vaccine was diluted by 1:100,000 to a target threshold cycle (C_t_) of 35, the A Argentina vaccine was diluted by 1:10,000 (C_t_ 28), and A_24_ Cruzeiro was diluted by 1:1000 (C_t_ 22). In addition, a tenfold dilution series of an O_1_ Manisa/O-3039 vaccine was prepared from 1:100 to 1:1,000,000 in fetal bovine serum (FBS). FBS was also used for the negative samples. Multiple replicates were included for all samples of the serotype O dilution series, but the SAT2, A Argentina, and A_24_ vaccines were represented by only one sample. From the dilution levels 1:100, 1:1000, and 1:10,000 of the O_1_ Manisa/O-3039 vaccine, three identical aliquots were provided. Of the samples with lower RNA concentrations (dilutions 1:100,000 and 1:1,000,000), a total of four aliquots were provided. Each panel included four aliquots of FMDV-free FBS (see Table 1). 

In the 16 samples of the serology panel, antibodies against FMDV were to be detected by ELISA. Three different sera were provided undiluted. One was obtained from a bovine vaccinated against FMDV A22 Iraq and subsequently infected with A Iran 96. It was collected on day 32 post infection. Another bovine serum was collected from an animal infected with FMDV A Iran 99 28 days after infection. To include a different host species, the serum of a goat infected with FMDV SAT2 Egypt 2014 was used. This serum was collected after 21 days. 

In addition to the undiluted sera, a dilution series of an antibody-positive serum in FBS was prepared. Serum from a bovine infected with FMDV O_1_ Manisa was collected on day 21 post infection and diluted, as shown in Table 2. Two identical replicates of each dilution step except 1:8 were included in the panel. The 1:8 dilution was represented by four replicates, FMDV antibody-negative serum was included three times, and there was one aliquot of each of the undiluted sera (see Table 2). All FMDV sera were tested to confirm the absence of detectable FMDV RNA and were heated to 56 °C for two hours to inactivate any residual virus.

Each sample in each of the 43 panels was randomly assigned a unique sample number. The sample IDs shown in the tables were not disclosed to the participants. The participants were told which samples were part of the virology or serology panel but were not made aware of the identity of any of the samples or the number of replicates per sample group. Even though the samples were not infectious, the laboratories were encouraged to treat them as field samples and to process them the same way as they would a routine submission.

The sample panels were shipped unrefrigerated to the participating laboratories. Most participants received the samples the following day, and the remainder received them on the second day after dispatch. To confirm that unrefrigerated shipping did not interfere with the analysis, additional virology and serology panels were retained at the NRL. One set was stored at ambient temperature and another at −20 °C. The panels stored at ambient temperature were analyzed after 5 days and the results were compared to the panels that had been kept frozen.

A total of 23 laboratories took part in the proficiency test, including state and federal veterinary diagnostic laboratories in Arnsberg, Aulendorf, Bad Langensalza, Detmold, Dresden, Erlangen, Fellbach, Frankfurt (Oder), Freiburg, Gießen, Hamburg, Hannover, Karlsruhe, Koblenz, Krefeld, Kronshagen, Leipzig, Neumünster, Oberschleißheim, Oldenburg, Rostock, Saarbrücken, and Stendal. All participants are accredited by DAkkS, the national accreditation body for the Federal Republic of Germany. 

It was left up to the participants to decide whether they wanted to receive only the virology or serology panel or both. Virological testing was performed by all participants and all used the FMDV 3D RT-qPCR assay [18] recommended by the OIE and the EU reference laboratory for FMD. Furthermore, 12 laboratories also used an IRES assay [19] that had in the past been recommended by the NRL but is now being phased out. Serological testing was carried out by 19 participants using the ID Screen FMD NSP Competition ELISA. This kit is currently held in the national FMD diagnostics bank, which was established to ensure the supply of serological tests for Germany in case of an outbreak. The most recent tender for the bank was awarded to ID Vet in 2018. For use in the proficiency test, one test kit was provided free of charge to each state laboratory that opted to receive the serology panel. 

In addition to testing the provided samples, the laboratories were asked to voluntarily report the number of virological and serological exclusion tests for FMD for each of the preceding four years (2017–2020) and provide details about their exclusion testing scheme. To verify the diagnostic specificity of the ELISA, the laboratories were requested to use the remaining reactions from the provided kits to test other samples from their routine caseload. 

## 3. Results

### 3.1. Detection of FMDV Genome by RT-qPCR

#### 3.1.1. 3D-OIE Assay 

Testing of the panels stored at the NRL confirmed the stability of the samples in the virology panel during shipping at ambient temperature. Even after 5 days at ambient temperature, C_t_ values had only increased by 1.3 on average compared to the frozen samples. 

**Figure 1:**
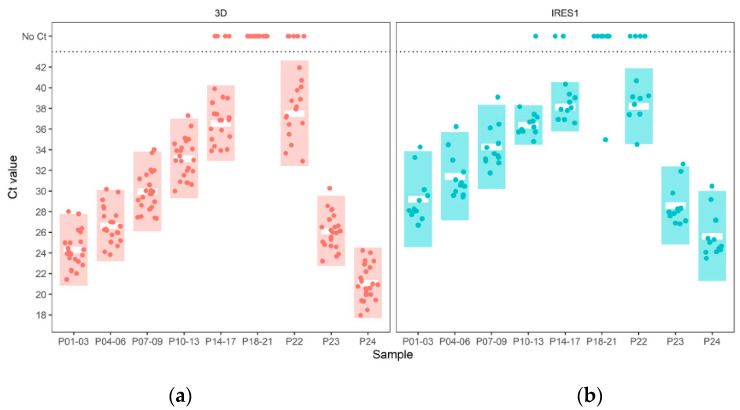
PCR results. The mean value of all results of each participant for a group of samples is shown as a single filled circle. The mean of means for all participants for each group of samples is represented by a white bar. The colored columns indicate the twofold standard deviation of the mean of means. (**a**) 3D-OIE assay; (**b**) IRES1 assay. See Table S1 in the Supplemental Material for the raw data summarized in this figure.

Up to a dilution of 1:10,000 (P07–P09; see Figure 1), all samples were correctly identified by all 23 participants using the 3D-OIE assay. At the next higher dilution of 1:100,000 (P10–P13), all but two laboratories were still able to identify all positive samples, and all laboratories returned a positive result for at least one of the four replicates of this dilution. At the 1:1,000,000 dilution (P14–P17), 60% of participants obtained positive results for all samples, 16% detected at least out of the four replicates as positive, but another 16% did not return a positive result for any of the four replicates.

Similarly, the more strongly positive samples P23 and P24 were detected reliably by all participants. Sample P22, which was the weakest sample in the virological panel, was correctly identified by 76% of the laboratories. All negative samples were correctly identified by all participants. Overall, the best results in terms of low C_t_ values and high clinical sensitivity were achieved with manual RNA extraction using the QIAmp Viral RNA Kit (QIAGEN, Venlo, The Netherlands) or automated extraction using the NucleoMag VET Kit (Macherey-Nagel, Düren, Germany) with a KingFisherFlex magnetic particle processor (Thermo Fisher Scientific, Waltham, MA, USA), followed by RT-qPCR with the SuperScript III One-Step RT-PCR System (Thermo Fisher Scientific).

##### 3.1.2. IRES1 Assay

The results obtained by the 12 laboratories using the IRES1 PCR were roughly similar to the 3D-OIE assay, but with reduced sensitivity, especially at the higher dilutions. Up to and including the 1:10,000 dilution (P07–P09), all positive samples were detected by all participants. Starting at the 1:100,000 dilution (P10–P13), however, some laboratories could no longer correctly identify some or all of the replicates. At the 1:1,000,000 dilution (P14–P17), only 23% of the participants returned positive results for all four replicates. The negative samples were identified correctly, with the exception of one laboratory, which evaluated one of the four replicates as positive. 

##### 3.1.3. Comparison between Assays 

To analyze the spread of the PCR results obtained by the participants, the two-fold standard deviation was calculated (see Table 3). There is a clear difference between the two PCR assays. With the 3D-OIE assay, only 9% of all results returned by the participants lie outside this range, compared to 13% with the IRES1 assay. (Negative results are included in this calculation).

#### 3.2. Detection of FMDV Antibodies Using the ID Screen FMD NSP Competition ELISA

Testing of the panels stored at the NRL confirmed the stability of the samples in the serology panel during shipping at ambient temperature. On average, after 5 days of storage, the S/N% ratios between the unrefrigerated and frozen samples differed by 2.1 percentage points, which had no impact on the interpretation of the ELISA. 

**Figure 2:**
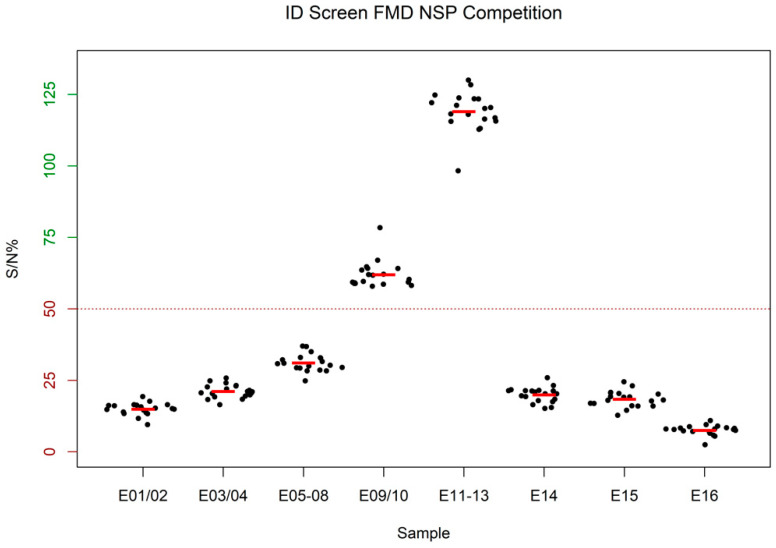
Results of the ID Screen FMD NSP Competition ELISA. The mean value of all results of each participant for a sample group is shown as a single filled black circle. The mean of means for all participants for each sample group is represented by a red bar. Samples with an optical density less than or equal to 50 % of the optical density of the negative control (S/N% ≤ 50) are considered positive. See Table S2 in the supplemental material for the raw data summarized in this figure.

All 19 participants receiving the serology panel correctly identified all samples (see Figure 2). All negative samples were also correctly evaluated as negative. A strong positive result was obtained for the 1:2 dilution (E01/02). With increasing dilution, the signal becomes weaker until it is finally below the cut-off at 1:32 (E09/10).

#### 3.3. Additional Specificity Data for the FMDV NSP Antibody ELISA 

Eleven of the participating laboratories provided ELISA data for additional samples from their routine caseload. These data were also collected using the ID Screen FMD NSP Competition ELISA. With nearly 50% of the samples, the dominant animal species was cattle, followed by 20% small ruminants and approx. 30% pigs (domestic and wild boar). Some zoo animals were represented, such as six samples from Bactrian camels, two samples from okapi and Kirk’s dik-dik, and one sample from a bison. A total of 2040 measurements were submitted, of which 2028 were negative and 12 were positive (Details on the positive samples can be found in Table S3 in the supplemental material). Ultimately, all positive results were considered false positives, and the overall specificity of the ELISA was calculated as 99.41%. 

#### 3.4. Exclusion Testing for FMD

Since Germany has been free of FMD for over 30 years, the disease has become increasingly obscure for many farmers and veterinarians. There are a wide variety of infectious and non-infectious conditions that can cause clinical signs similar to FMD, such as stomatitis, foot lesions, or lameness. When FMD cannot be ruled out based on the clinical presentation alone, a laboratory investigation is required. In Germany, samples can be submitted for FMD exclusion testing without any restrictions being placed on the farm. In order to get an indication of the acceptance of the exclusion testing scheme, the participating laboratories were asked to report their activities for the previous four years (see Table 4). 

In 45% of the laboratories, exclusion tests for FMD are only carried out when specifically requested by the client. The remaining 55% also initiate tests on their own accord, e.g., if the case history includes clinical signs congruent with FMD. Of the submissions for the exclusion testing scheme, 44% come from official veterinarians, followed by 32% from practitioners and 24% from other facilities, such as universities or border control points at airports, which submit confiscated animal products. The costs are usually borne by the laboratory or by the state animal disease fund. If the samples come from necropsies carried out at the regional laboratories, the cost for laboratory tests is included in the fee to be paid by the animal owners. No fees are charged for necropsies mandated by an official veterinarian. 

## 4. Discussion

### 4.1. Virology

Overall, all participating laboratories successfully detected FMDV RNA by RT-qPCR in all sample groups. In some laboratories, problems became apparent at lower RNA concentrations. This was the first national FMD ring trial that included many weakly positive samples with several replicates per dilution. In previous ring trials in Germany, smaller sample panels were used, and the participants were asked to prepare their own non-blinded serial dilutions [20]. It is expected that highly positive samples, such as fresh lesion material, can be reliably detected by RT-qPCR in any laboratory. However, not all samples conceivably collected from a suspect case of FMD contain high levels of RNA. As with many other contagious diseases, the amount of virus and thus viral RNA excreted is low early after infection, rises to a peak roughly coinciding with pronounced clinical signs, and then falls as the host’s immune response increases [21]. Samples taken very early or very late or samples from old lesions may therefore contain only small amounts of FMDV RNA. It is open to debate, however, what level of analytical sensitivity is necessary to ensure adequate diagnostic sensitivity. Does it need to be pushed to the limit in a free country? Does a false negative result for a single sample with a low viral load necessarily lead to a significant delay in the detection of an outbreak? When considering this question, it is first necessary to look at the situation in the field. The veterinarian is usually consulted only in advanced clinical cases or when several animals are already affected. Even legal mandates intended to promote early detection rely on clusters of clinical cases within 7-day periods [13]. It is thus unlikely that samples are taken too early in infection. Sampling too late or from mature lesions with low virus load can be avoided by a comprehensive examination of the entire epidemiological unit rather than only the most obviously affected animal. Increasing the awareness of FMD among farmers and practitioners and encouraging submissions to exclusion testing programs will likely be more beneficial than attempting to further increase the analytical sensitivity of already very sensitive diagnostic tests. In any case, it is important to conduct a thorough assessment of the costs, benefits, and risks of diagnostic tests in light of their intended application. The relative importance of sensitivity and specificity can vary greatly depending on the purpose of the test, e.g., exclusion testing in a free country versus surveillance to demonstrate freedom from FMDV infection after an outbreak. 

As it was, the negative samples included in the virology panel were intended to detect cross-contamination between positive and negative samples during sample processing as well as a contamination of the laboratory environment by PCR products or positive control material. Future proficiency trials, however, should also include material from other vesicular disease viruses such as Senecavirus A or vesicular stomatitis viruses to better demonstrate the specificity of the RT-PCR assays used by the participating laboratories.

### 4.2. Serology

All participating laboratories correctly identified all positive samples, demonstrating adequate sensitivity of NSP antibody detection. Likewise, all negative samples were detected as such. However, as with the virology panel, the material used for the negative samples, commercial fetal bovine serum, was not ideal. The ELISA kits in the German diagnostic reagent bank are intended to be used for post-vaccination surveillance. To make sure that there will not be an unacceptably high rate of false-positive results when testing a vaccinated population, future proficiency trials should include sera from vaccinated but not infected animals as the negative sample set.

Beyond the negative samples provided by the NRL, the participating laboratories tested samples from their routine caseload. All samples originated from Germany. In this sample set, the specificity of the ELISA was calculated to be 99.41%. In the calculation, all positive results were considered false positives. Five of the twelve initially positive samples were negative in further tests. Another five were strongly hemolytic and bacterially contaminated plasma samples collected from wild boar carcasses, where the positive ELISA result was considered unreliable due to the poor sample quality. While there was no resolution or explanation for the two remaining positives, they were also considered to be spurious. This assumption is grounded in the fact that Germany has been free of FMD since 1988, and no outbreaks of FMD were reported anywhere in Europe during or after the study period. 

In general, the quickest and easiest way to resolve a false positive result is to repeat the test after heat inactivation of the samples at 56 °C for 30 min, which can reduce many unspecific reactions [22]. If this does not succeed, it can be helpful to re-test the samples with a different assay [23]. The choice of the test to be used in this case is difficult, though. It has been shown that the two most common competitive NSP antibody ELISAs, ID Vet NSP Competition and PrioCHECK FMDV NS, can be used interchangeably [22]. So how does one interpret a positive result in one test and a negative result in the other? In this situation, neutralization tests are also unlikely to be useful since they are serotype-specific and less sensitive than most ELISAs. 

If the problem cannot be resolved with the available sample material, it may be advisable to obtain a fresh sample from the animal in question (and possibly other animals from the same herd), which also avoids issues related to low sample quality. Bacterial contaminants can produce antibody-binding proteins that cause unspecific reactions [24]. 

It is possible, however, that the positive reaction will persist even with repeated sampling. For swine vesicular disease virus, between 1 and 3 of 1000 pigs are so-called “singleton reactors”, which are serologically positive in the absence of any evidence of previous infection [25]. Similar phenomena were described for other viruses, such as bovine herpesvirus, where they are caused by cross-reactions between related viruses [26].

Ultimately, the final interpretation of each case must consider all available clinical, serological, virological, and epidemiological evidence. 

### 4.3. Exclusion Testing

In the exclusion testing scheme, RT-qPCR plays a much larger role than NSP antibody ELISAs (1581 vs. 232 tests between 2017 and 2020) because FMD exclusion tests are usually prompted by acute clinical observations or suspicious post-mortem findings. Only a few hundred samples per year are submitted for FMD exclusion testing in Germany, which is very low compared to the number of cattle and pigs (approx. 11.3 million and 26 million) [27]. Most samples are still sent in by official veterinarians and not by practitioners. Here, further outreach could create more awareness of how important exclusion testing is and that there are no adverse consequences for the animal owner.

## 5. Conclusions

All participating laboratories are fully proficient in FMD diagnostics. FMDV antigen detection by RT-qPCR is routinely used for FMD exclusion tests. The number of exclusion tests is at an acceptable level but should be further increased to safeguard the freedom from FMD in Germany and the EU.

## Figures and Tables

**Table 1:** Sample panel for virological testing.

ID	Content	ID	Content	ID	Content
P01	O 1:100	P09	O 1:10,000	P17	O 1:1,000,000
P02	O 1:100	P10	O 1:100,000	P18	FMDV-negative serum
P03	O 1:100	P11	O 1:100,000	P19	FMDV-negative serum
P04	O 1:1000	P12	O 1:100,000	P20	FMDV-negative serum
P05	O 1:1000	P13	O 1:100,000	P21	FMDV-negative serum
P06	O 1:1000	P14	O 1:1,000,000	P22	SAT2 1:100,000
P07	O 1:10,000	P15	O 1:1,000,000	P23	A Argentina 1:10,000
P08	O 1:10,000	P16	O 1:1,000,000	P24	A_24_ Cruzeiro 1:1000

**Table 2:** Sample panel for serological testing.

ID	Content	ID	Content	ID	Content
E01	Serum O_1_ 1:2	E06	Serum O_1_ 1:8	E11	FMDV antibody-free serum
E02	Serum O_1_ 1:2	E07	Serum O_1_ 1:8	E12	FMDV antibody-free serum
E03	Serum O_1_ 1:4	E08	Serum O_1_ 1:8	E13	FMDV antibody-free serum
E04	Serum O_1_ 1:4	E09	Serum O_1_ 1:32	E14	Serum A_22_
E05	Serum O_1_ 1:8	E10	Serum O_1_ 1:32	E15	Serum A Iran 99
				E16	Serum SAT2

**Table 3:** Overview of the PCR results. Indicated for each sample group are the mean value and the twofold standard deviation of the results submitted by all participants.

P-No.	Dilution	C_t_ Value 3D-OIE		C_t_ Value IRES1		∆C_t_ Value IRES1—3D-OIE
P01–P03	1:100	24.3	±3.5	29.2	±4.7	4.9
P04–P06	1:1000	26.6	±3.4	31.4	±4.3	4.8
P07–P09	1:10,000	30.0	±3.9	34.3	±4.1	4.3
P10–P13	1:100,000	33.1	±3.8	36.4	±1.8	3.3
P14–P17	1:1,000,000	36.6	±3.7	38.2	±2.4	1.6
P18–P20	Negative	Negative		Negative		/
P22	Undiluted	37.5	±5.1	38.2	±3.7	0.7
P23	Undiluted	26.1	±3.4	28.6	±3.8	2.5
P24	Undiluted	21.1	±3.4	25.6	±4.4	4.5

**Table 4:** Total number of FMD tests at regional laboratories from 2017 to 2020.

Test	Year	Samples
FMDV RT-qPCR	2017	607
	2018	378
	2019	385
	2020	211
FMDV NSP antibody ELISA	2017	37
	2018	82
	2019	75
	2020	38

## Data Availability

All quantitative data from the study are available in the supplemental material.

## Supplementary Materials

The following supporting information can be downloaded at: https://www.mdpi.com/article/10.3390/v14051098/s1, Table S1: Virology data returned by the participants. Table S2: Serology data returned by the participants. Table S3: Field samples with false positive results in the NSP ELISA.
